# Bilateral superior and inferior thyroid artery embolization for massive thyroidectomy site bleeding: A case report

**DOI:** 10.1016/j.radcr.2025.09.014

**Published:** 2025-10-04

**Authors:** Zineb Essolaymany, Zineb Yammouri, Hajar Ouazzani, Ismail Chaouche, Amal Akammar, Nizar El Bouardi, Badreddine Alami, Moulay Youssef Alaoui Lamrani, Meryem Boubbou, Mustapha Maâroufi

**Affiliations:** a Radiology Department, CHU Hassan II, Fez, Morocco; b Mother and Child Radiology Department, CHU Hassan II, Fez, Morocco; c Faculty of Medicine and Pharmacy of Fez, University Sidi Mohammed Ben Abdellah, Fez, Morocco

**Keywords:** Thyroidectomy, Massive bleeding, Thyroid artery, Embolization, Coiling

## Abstract

Thyroidectomy is a well-described procedure involving partial or total excision of the thyroid gland. It can be associated with some worrisome and dangerous postoperative complications, including postoperative hematoma. Embolization of the thyroid arteries is of great benefit in cases of persistent or recurrent bleeding in the thyroid bed, but is very rarely described in the setting of a post-operative hematoma. We describe a rare case of a patient admitted for a massive thyroidectomy site bleeding who underwent bilateral embolization of superior and inferior thyroid arteries using micro-coils, after unsuccessful surgical attempts at hemostasis.

## Introduction

Thyroidectomy is a widely performed surgical procedure, indicated for malignant neoplasms of the thyroid (such as papillary, follicular, or medullary carcinoma), benign conditions including multinodular goiter and recurrent nodular disease, as well as functional disorders such as Graves’ disease, toxic multinodular goiter, and autonomously functioning thyroid adenoma that are refractory to medical management [1,2]. It can be associated with serious postoperative complications, including laryngeal nerve damage, hypocalcemia, hematoma and infection [3]. Post-thyroidectomy hematomas are rare, but potentially life-threatening. Computed Tomography angiography (CTA) is helpful in identifying the origin of bleeding and defining arterial anatomy. Arterial embolization is recommended in cases of persistent bleeding after conservative or surgical management of the hemorrhage [4]. It can be used as an effective hemostatic treatment, particularly in cases of active hemorrhage with contrast extravasation on CTA. Nevertheless, the thyroid gland demands a different approach than the other organs because of its complex, exceptionally rich and highly variable blood supply from both carotid and subclavian branches, its anatomical relationship to vital neurovascular structures, and the risk of precipitating hormone release during vascular compromise. Consequently, reports on thyroid artery embolization remain scarce, as revision surgery continues to represent the standard hemostatic treatment in such cases. This case report demonstrates our embolization approach for a massive and recurrent cervical bleeding after total thyroidectomy, following unsuccessful surgical hemostasis and involving both the superior and inferior thyroid arteries, a scenario that has been rarely documented in the literature.

## Case presentation

A 68-year-old male patient was admitted to the Ear-Nose-Throat (ENT) emergency department to undergo an exploratory surgery for a massive post-thyroidectomy cervical hemorrhage. Anamnesis revealed that the patient had undergone a total thyroidectomy as treatment of goiter in another facility 3 months prior to his current admission. The patient was not receiving any form of anticoagulation at presentation. The post-operative course was marked by the onset of swelling and progressive pain in the anterior neck 3 weeks later, related to a voluminous hematoma in the thyroid bed, leading to dyspnea and dysphonia, requiring urgent surgical drainage.

Clinical assessment revealed a pale patient with mild tachycardia. His heart rate was 110 beats per minute. Blood pressure was stable at 120/60 mmHg, and oxygen saturation level was at 95%. He had a cervical swelling with an open thyroidectomy site incision, still bleeding, resulting in airway compromise, with stridor and slight dyspnea. Biological tests showed severe anemia with a hemoglobin level at 7 g/dL. The platelets count was at 360,000/mm³, the International Normalized Ratio (INR) at 1 and the C reactive protein at 17 mg/L.

**Fig. 1:**
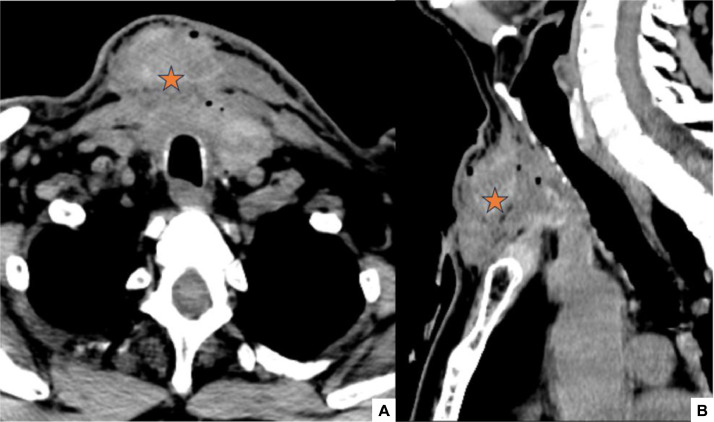
Axial (A) and Sagittal (B) pre-contrast cervical CT images on admission : Large and heterogenous hematoma in the anterior neck space, containing air bubbles (asterisk).

On the first line of investigations, a cervical Computed Tomography (CT) was performed, demonstrating a large and heterogenous hematoma in the anterior neck space (Fig. 1), with no active bleeding after contrast administration.

**Fig. 2:**
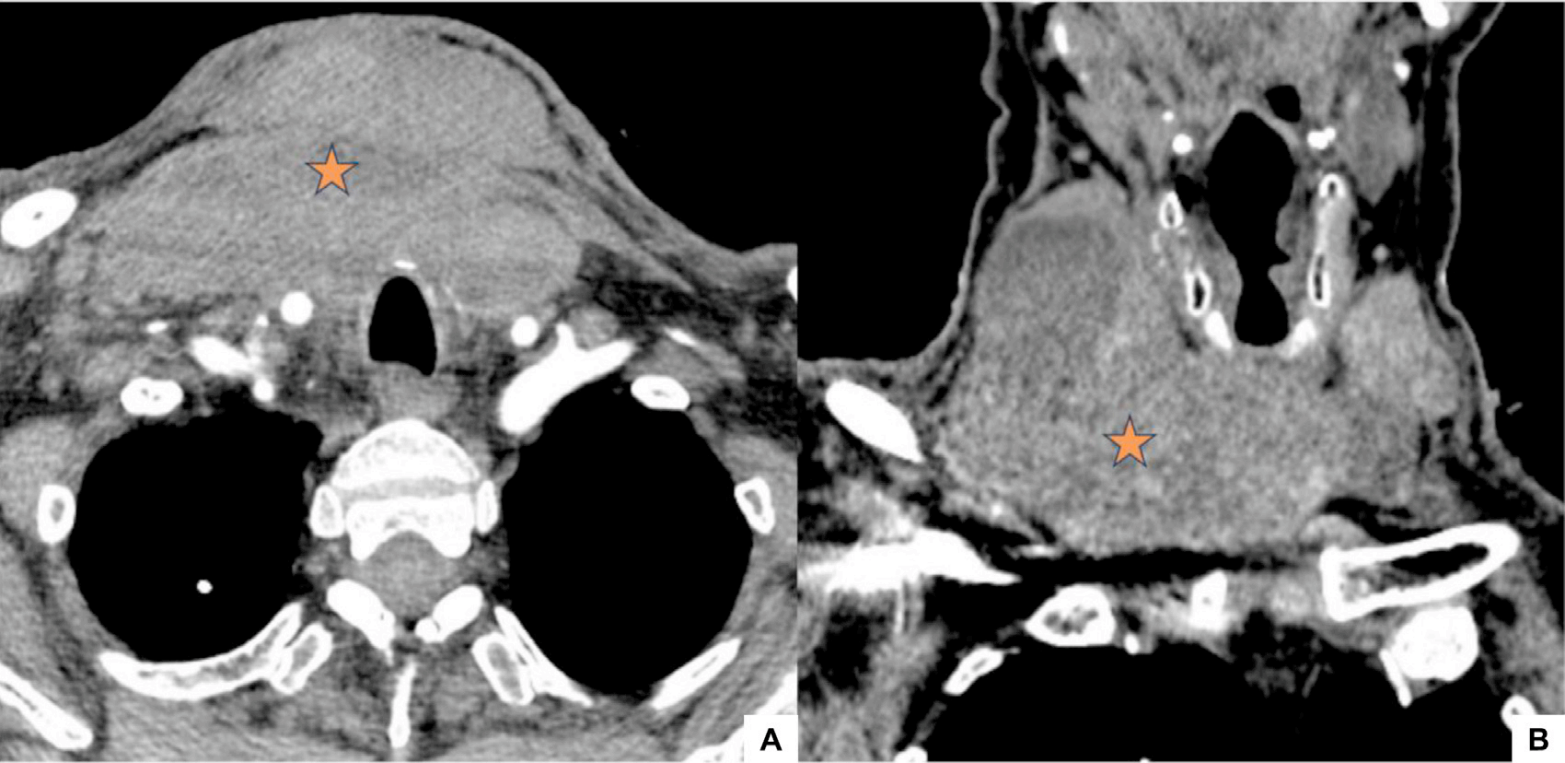
Axial (A) and Coronal (B) cervical CTA images after revision surgery: Significant increase in hematoma volume (asterisk), causing slight tracheal deviation, with no signs of active bleeding in the hemorrhage site.

The patient received red blood cell (RBC) transfusion and was admitted to the operating room (OR) for an exploratory cervicotomy, which identified an organized cervical hematoma with low-flow bleeding. The patient underwent a surgical drainage of the hematoma with ligation of superficial veins in the thyroid bed. He was put on prophylactic antibiotics, tranexamic acid and analgesics. The post-operative course was marked by the persistence of the bleeding and the patient was readmitted to the OR 72 hours later for revision surgery, with vessel ligation and placement of biologic glue and cellulose-based hemostatic agents. Coagulation tests showed a normal fibrinogen level at 3.23 g/L and a normal bleeding time measured at 2.08 minutes. Factor VIII and von Willebrand Factor (vWF) levels were also within the normal range, at 200% and 89%, respectively. His follow-up cervical CTA demonstrated a significant increase in hematoma volume, causing slight tracheal deviation. There was no active bleeding in the hemorrhage site (Fig. 2).

**Fig. 3:**
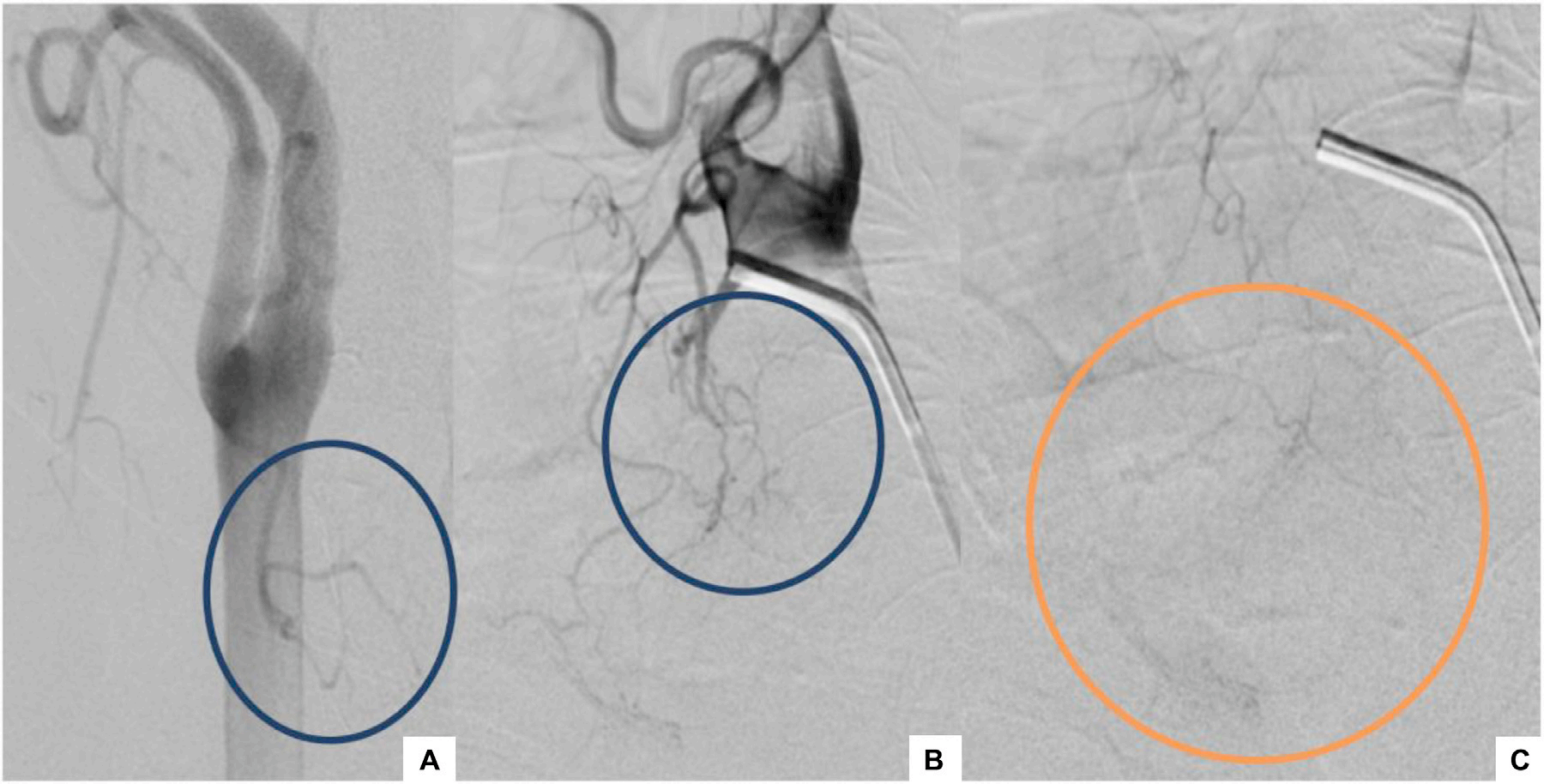
Angiographic images: Right (A) and left (B, C) external carotid angiography revealed a discreetly dilated right (a) and left (B) STA (blue circles) with hyperemia in the left thyroid bed (orange circle).

Due to the failure of surgical hemostasis, angiography was performed as a secondary diagnostic and therapeutic procedure. The procedure was performed in an angiography unit under local anesthesia and fluoroscopic monitoring. Following right femoral puncture, a 5-Fr angled catheter was advanced into the aorta via a sheath positioned in the femoral artery, along with a stiff-angled guidewire. Right and left thyrocervical trunks angiography revealed no absolute evidence of bleeding. Right and left external carotid angiography revealed a discreetly dilated superior thyroid artery (STA) feeding multiple vermicular arteries, with hyperemia in the left thyroid bed; it didn’t show any signs of active bleeding (Fig. 3).

**Fig. 4:**
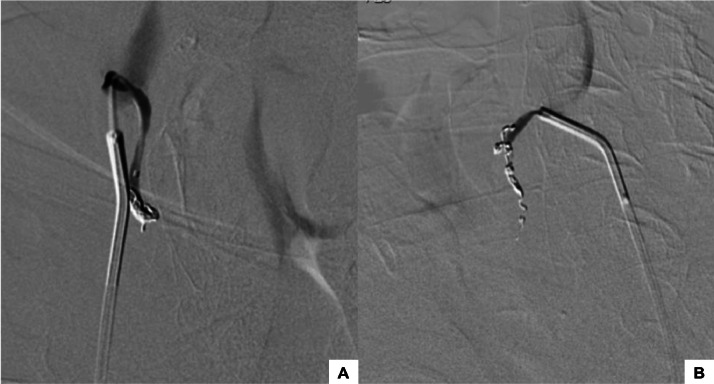
Angiographic images: Post-embolization angiogram revealed truncal occlusion of the right (A) and left (B) STA.

Given the presence of subtle angiographic hyperemia, the patient’s ongoing clinical bleeding, and the absence of other identifiable targets, empirical bilateral embolization of the STA was considered a reasonable strategy to mitigate the risk of further hemorrhage. A microcatheter (Echelon 18 Microcatheter, 2.4 F, ev3 Inc) was navigated using a microguide wire (Traxcess 14 Guidewire, 0.014 inch, Terumo Corporation) allowing for superselection of the right STA, and embolization was performed using 3 coils (Axium Prime, ev3 Inc), measuring 2.5 mm x 4 cm, 1.5 mm x 2 cm and 1 mm x 2 cm. The same procedure was performed on the left side, using 1 coil, measuring 3 mm x 8 cm. Post-embolization angiogram showed truncal occlusion of the STA (Fig. 4).

**Fig. 5:**
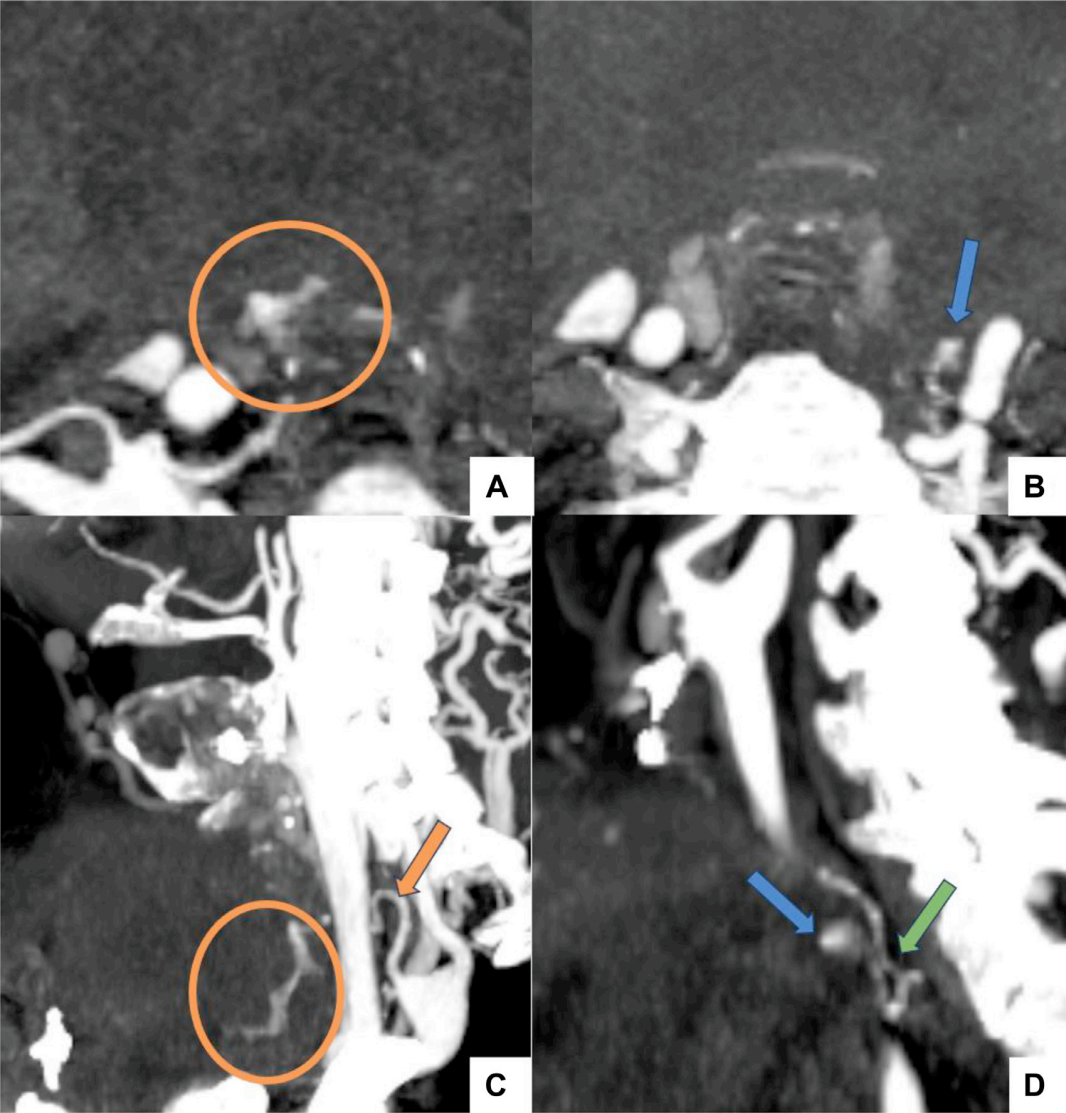
Axial (A, B) and Oblique Sagittal (C, D) cervical CTA images: Large cervical hematoma with an arterial blush (orange circle) arising from the right ITA (orange arrow), confirming an active bleeding (A, C). A small pseudoaneurysm (blue arrow) arising from the left ITA (green arrow) (B, D).

The bleeding persisted, but considering the risk of procedure-related complications, it was decided to maintain conservative management for the hematoma, and a third cervical CTA was performed 7 days later to look for a bleeding focus. It revealed a large cervical hematoma with an arterial blush arising from the right inferior thyroid artery (ITA), confirming an active bleeding (Figs. 5A and C). It revealed a small pseudoaneurysm arising from the left ITA as well (Fig. 5, Fig. 5).

**Fig. 6:**
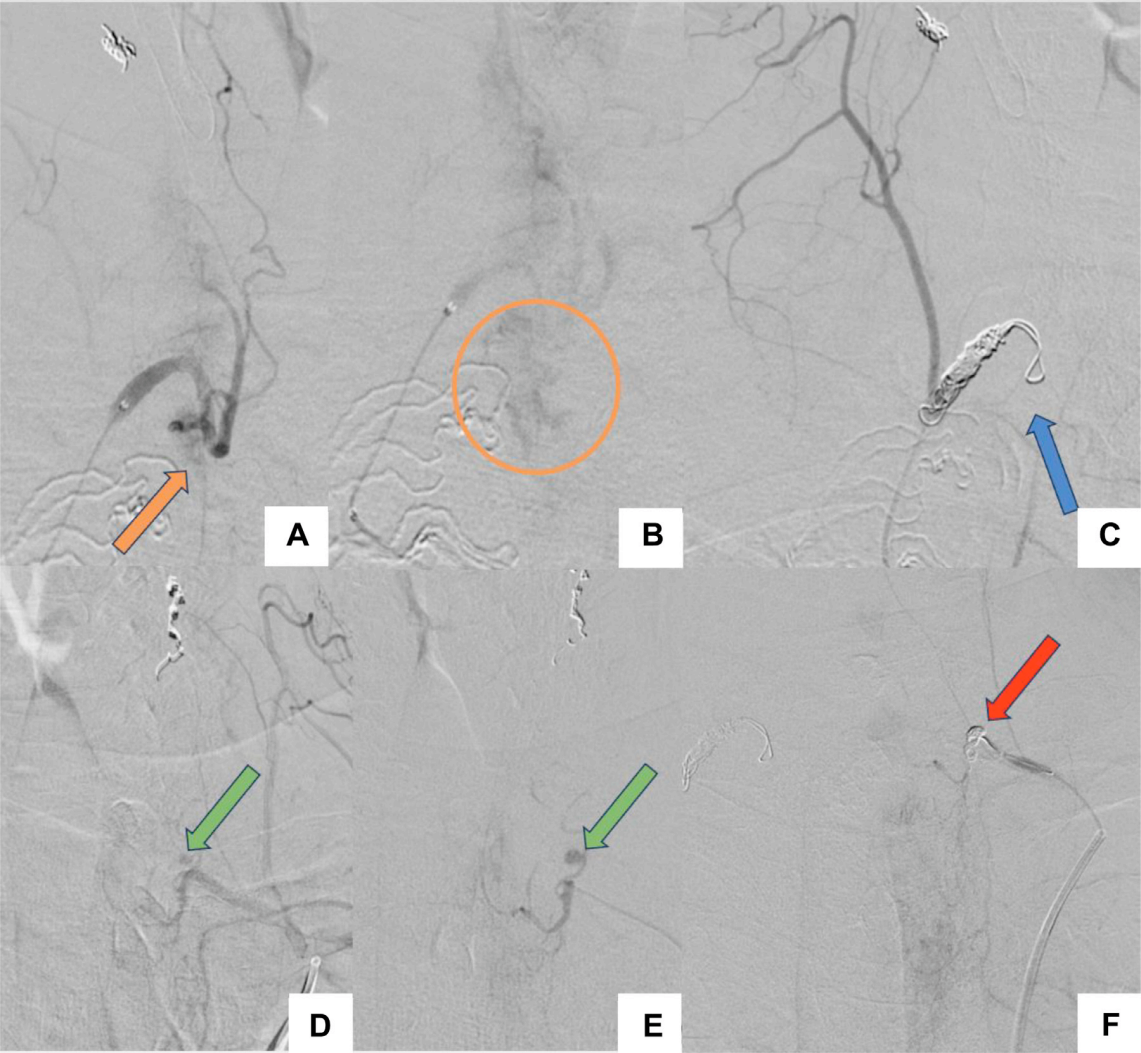
Angiographic images: The bleeding focus (orange arrow and circle) was identified on selective angiography of the right inferior thyroid artery (A, B). Embolization was performed using 04 coils (blue arrow) (C). Left inferior thyroid artery angiography revealed a small contrast-filled pouch (green arrow) compatible with a pseudoaneurysm (D, E). Embolization was performed using 1 coil (red arrow) (F).

Due to the progressive expansion of the hematoma, the patient was readmitted to the Angiography Suite. The ITA was identified as an anteriorly-projecting branch of the subclavian artery. It was catheterized with a microcatheter (Rebar 18 Microcatheter, 2.4 F, ev3 Inc), which was navigated using a microguide wire (Traxcess 14 Guidewire, 0.014 inch, Terumo Corporation), allowing for superselection of the right ITA and revealing the bleeding focus, which matched the CT image (Figs. 6A and B). Occlusion of the right ITA was performed using 4 coils (Axium Prime, ev3 Inc), measuring 5 mm x 10 cm, 4 mm x 8 cm, 3 mm x 4 cm and 4 mm x 8 cm.

Selective angiography of the left ITA, using the same technique, revealed a small, saccular contrast-filled pouch, measuring approximately 04 mm, consistent with a pseudoaneurysm, that matched the CT image (Fig. 6, Fig. 6). After superselection of the left ITA using a microcatheter (Rebar 18 Microcatheter, 2.4 F, ev3 Inc), navigated on a microguide wire (Traxcess 14 Guidewire, 0.014 inch, Terumo Corporation), for sufficient support, embolization was performed using 1 coil (Axium Prime, ev3 Inc), measuring 4 mm x 10 cm.

Angiographic control showed occlusion of the ITA on both sides (Fig. 6, Fig. 6).

Both procedures were performed without systemic heparinization due to the patient's recent surgery and elevated bleeding risk. Additionally, femoral access was used, further supporting the decision to avoid heparin. No vasospasm or contrast reflux was observed during the angiography.

Successful embolization was achieved, the hemorrhage completely stopped 3 days later. After surgical evacuation of the cervical hematoma, the patient was discharged in a stable condition. He will be in close follow-up every month in the ENT department.

**Fig. 7:**
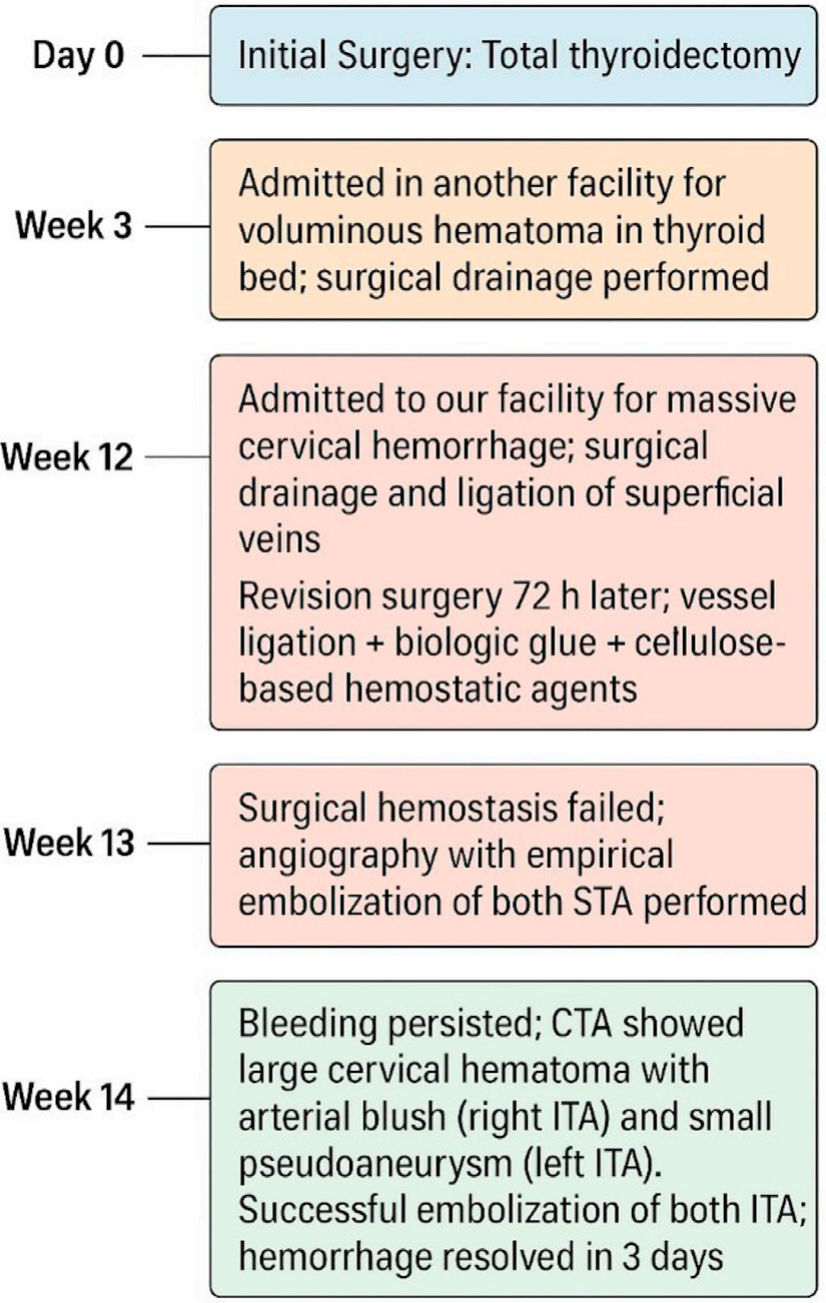
Timeline of hemorrhagic events and interventions.

The following timeline summarizes the postoperative course of our patient. It highlights the sequence of hemorrhagic events, diagnostic evaluations, and therapeutic interventions from the time of the initial surgery to the resolution of bleeding (Fig. 7).

## Discussion

The delicate anatomy of the anterior region of the neck, the proximity of vital structures and the narrow working space make thyroidectomy a difficult procedure to perform effectively and safely.

Although infrequent, postoperative hematoma can pose a life-threatening risk following thyroidectomy, leading to airway obstruction and respiratory distress. The incidence of cervical hematoma following thyroidectomy is ranging between 0.7 and 4.7% throughout different studies, with most cases occurring within the first 6-24 hours postoperatively [5]. Delayed postoperative bleeding, as observed in this case 3 weeks after surgery, is uncommon. Such delayed hemorrhage is often associated with pseudoaneurysm formation, which can develop secondary to vessel wall injury or incomplete hemostasis during the initial procedure. In the present case, imaging confirmed the presence of a pseudoaneurysm, highlighting the importance of considering this diagnosis when patients present with late-onset bleeding.

The etiopathogenesis of postoperative hematomas is multifactorial, and includes slipping of ligatures, reopening of previously cauterized veins and bleeding from residual thyroid parenchyma [6,7].

Few studies have examined risk factors of postoperative bleeding after thyroidectomy. High blood pressure is one of the most important ones, followed by administration of anticoagulants and coagulopathy. Male gender, older age, smoking, coexisting morbidities and hyperthyroidism have also been mentioned as risk factors for post-operative hematoma in several reports [6,7]. Infection is also a major reason for fatal post-operative bleeding occurring in patients who undergo thyroid operations [8].

When hemostasis cannot be achieved with manual compression and life-threatening bleeding is suspected, the management algorithm typically begins with immediate bedside evacuation of the hematoma to secure the airway, followed by urgent surgical re-exploration to achieve definitive hemostasis; in cases where the bleeding source remains unclear or surgical control is not feasible, an urgent diagnostic radiological workup may be indicated to guide interventional management. The multidetector CT is helpful in identifying the origin of the bleeding because it provides better image quality with rapid image acquisition and various multiplanar image reformations [9].

The thyroid is a richly vascularized gland. The STA usually arises from the external carotid artery just below the level of the greater cornu of the hyoid bone and enters the cervical fascia before giving anterior and posterior branches [10], that primarily supply the upper and anterior parts of the gland.

The ITA usually arises from the thyrocervical trunk and supplies the inferior and posterior parts of the gland [10]. The thyroidea ima artery is an uncommon variant, often associated with absent inferior thyroid arteries; it commonly arises from the brachiocephalic trunk, ascends on the anterior surface of the trachea and supplies both the trachea and thyroid [11]. CT helps the interventional radiologist to plan for the procedure and decrease the number of angiograms required to localize, not only the bleeding site, but also the target vessel by showing an irregular segment of the artery, a pseudoaneurysm or active contrast extravasation.

In the setting of hematomas due to STA or ITA rupture, severe hypovolemia could result from excessive hemorrhage in the cervical soft tissues and the superior mediastinum, with great risk of airway obstruction by the hematoma, requiring aggressive management. Surgical ligation is preferred in these cases of rupture, since airway maintenance can become troublesome due to the mass effect of the hematoma [9,12]. However, surgery carries risks of recurrent or phrenic nerve injury and surgical site infection, in addition of requiring general anesthesia. In contrast to surgical procedures, arterial embolization is a safer option with lower complication rates and shorter hospitalization [9]. It can target the bleeding focus and allows empirical embolization when the bleeding focus is not clearly identified on CTA or selective angiography, though it is not without inherent risks. Potential complications include non-target embolization, ischemia of adjacent tissues, and vascular injury, which must be carefully considered, particularly in anatomically complex regions. Thus, the choice between embolization and surgical management should be individualized, weighing the relative risks and benefits in the context of the patient’s clinical status and prior interventions. Embolization of the thyroid arteries has been described as an effective preoperative treatment of hyperfunctioning and plunging goiters [13], or as an alternative to thyroidectomy [14]. But in the setting of a post-thyroidectomy hematoma, very little is found in the literature.

Embolization techniques have evolved significantly over time and the variety of embolic agents has considerably increased. The choice of the embolic agent is an important decision that must be made before any embolization procedure [15]. The size of the vessel, the need of the downstream organ or tissue to remain viable after the procedure, and whether the occlusion should be temporary or permanent are 3 major keypoints that must be specified in order to choose the best agent to use [16]. Angiographic findings of vessel injury also play a part in this decision [9]. There is a whole range of embolic agents, with different advantages and a required level of expertise for effective use [15].

The most suitable embolic agents for occluding small arteries, such as thyroid arteries, are micro-coils and liquid agents. Cyanoacrylate liquid glues are usually used to control massive and active bleedings [16]. They are tissue adhesive materials that polymerize rapidly once exposed to an ionic environment such as blood, forming a cast of the targeted vessel [16], which is beneficial for massive hemorrhages that require urgent hemostasis. They offer the advantage of eliminating the risk of an associated hypocoagulability, that can be due to consumption of coagulation factors as a consequence of recurrent bleeding [17]. However, the risk of reflux in high-flow arteries, such as internal carotid arteries, makes their use perilous [17]. Micro-coils are best suited for complex anatomical situations owing to their controlled release [18]. We chose to use coils for embolization in this case due to their ability to provide precise mechanical occlusion, which is particularly important in situations where prior surgical interventions have failed or where distal non-target embolization poses a significant risk. Unlike liquid agents or particles, coils allow controlled deployment at the intended site, minimizing the chance of unintended ischemia in surrounding tissues. Additionally, the physical structure of coils is advantageous for achieving durable vessel occlusion in larger or tortuous vessels where liquid embolics may be less predictable [18].

## Conclusion

Although postoperative hematoma after thyroidectomy is uncommon, it can be potentially life-threatening due to hypovolemia and respiratory distress. Embolization of the thyroid arteries is rarely reported in the setting of a post-thyroidectomy cervical hematoma. We successfully performed bilateral embolization of superior and inferior thyroid arteries using coils, to control a massive post-thyroidectomy bleeding. This treatment option can be indicated in cases of recurrent and threatening hemorrhage after attempted surgical hemostasis, particularly in the presence of signs of active bleeding on CTA.

## Abbreviations

CTA, computerized tomography angiography; CT, computerized tomography; ENT, ear-nose-throat; INR, international normalized ratio; RBC, red blood cell; OR, operating room; vWF, von Willebrand factor; STA, superior thyroid artery; ITA, inferior thyroid artery.

## Patient consent

Written informed consent for the publication of this case report was obtained from the patient. A copy of the consent form is available for review by the Editor of this journal.
